# Infarto do Miocárdio Inferior Evoluído com Pseudoaneurisma do Ventrículo Esquerdo: Um Dilema Diagnóstico

**DOI:** 10.36660/abc.20200029

**Published:** 2020-05-12

**Authors:** Sónia Gomes Coelho, Clara F. Jorge, Pedro B. Carlos, Anne Delgado, Leopoldina Vicente

**Affiliations:** 1 Centro Hospitalar Cova da Beira EPE Department of Internal Medicine Covilhã Portugal Centro Hospitalar Cova da Beira EPE - Department of Internal Medicine, Covilhã - Portugal; 2 Centro Hospitalar Cova da Beira EPE Department of Cardiology Covilhã Portugal Centro Hospitalar Cova da Beira EPE - Department of Cardiology, Covilhã - Portugal

**Keywords:** Infarto do Miocárdio/complicações, Pseudoaneurisma, Ruptura Cardíaca, Ecocardiografia/métodos, Espectroscopia de Ressonância Magnética/métodos

## Introdução

O pseudoaneurisma (PA) do ventrículo esquerdo (VE) constitui uma complicação mecânica rara do infarto agudo do miocárdio (IAM).^1^ Resulta de ruptura miocárdica, em que o processo hemorrágico é contido pelo pericárdio aderente. Ocorre mais comumente na parede ventricular inferior e posterior, uma vez que a ruptura da parede anterior do ventrículo conduz habitualmente ao tamponamento cardíaco e morte imediata, enquanto que a face ínfero-posterior do coração se apoia sobre o diafragma, facilitando a contenção da cavidade ventricular pelo pericárdio.^1 - 3^ Os métodos de imagem são cruciais para estabelecer o diagnóstico. A ecocardiografia transtorácica (ETT) e transesofágica permite o diagnóstico definitivo em 26% e 75% dos casos, respectivamente.^1 , 2^ A ressonância magnética cardíaca (RMC) é útil no diagnóstico diferencial de PA e aneurisma do VE, com uma sensibilidade reportada de 100%.^2^ A presença de realce tardio pericárdico na RMC é um achado altamente sugestivo de PA do VE, podendo representar o efeito da passagem de sangue para o espaço pericárdico quando da ruptura miocárdica, com subsequente inflamação e fibrose pericárdicas.^1 , 2 , 4^

## Relato do Caso

**Figura 1:**
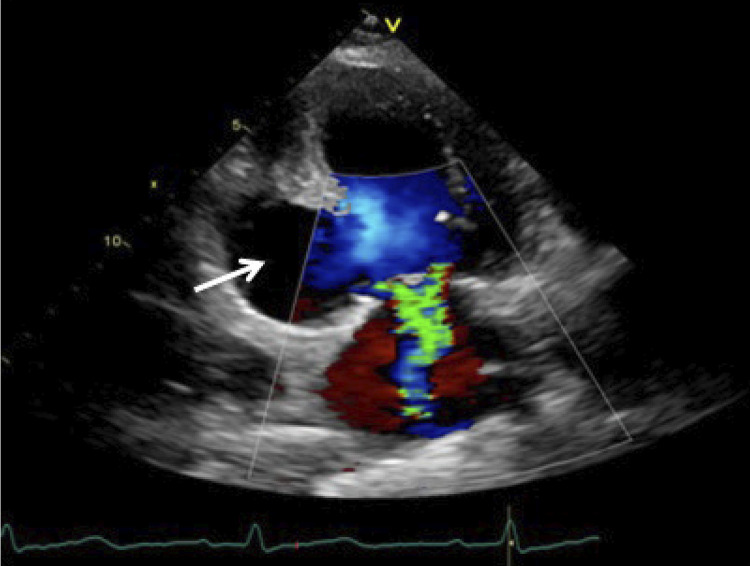
Ecocardiografia transtorácica (incidência apical 2 câmaras) evidenciando formação aneurismática (seta).

**Figura 2:**
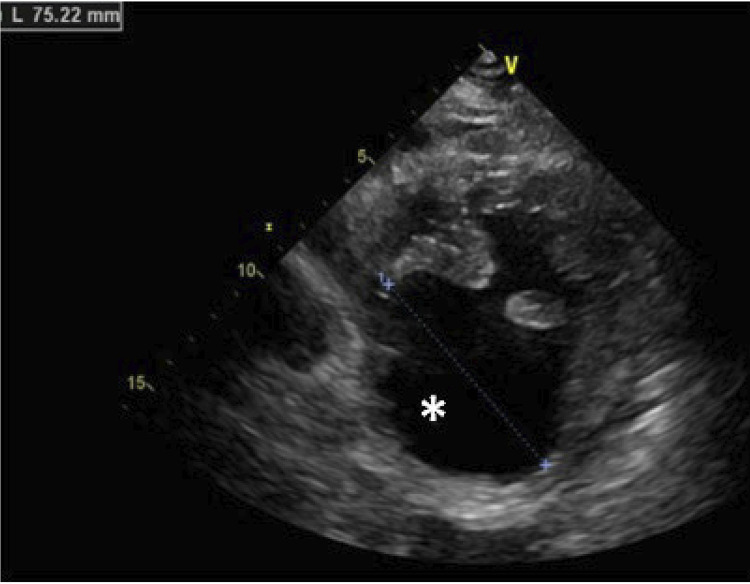
Ecocardiografia transtorácica (incidência eixo curto) sugestiva de pseudoaneurisma da parede inferior do ventrículo esquerdo (asterisco).

**Figura 3:**
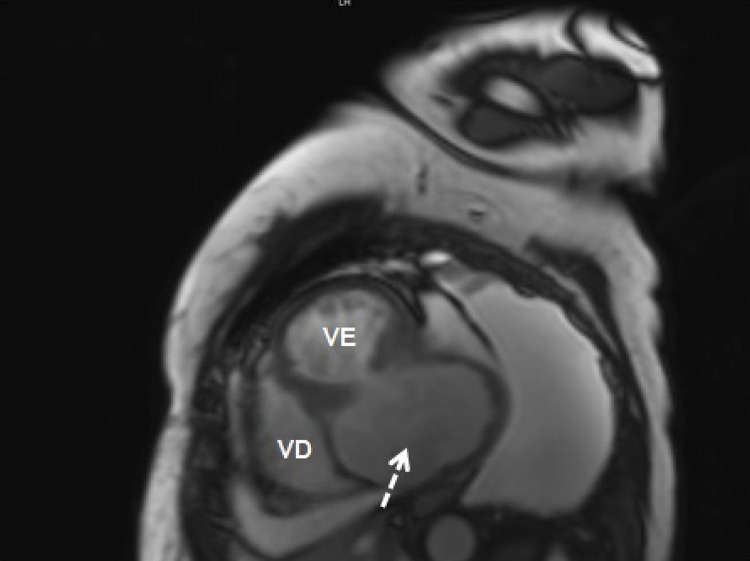
Ressonância magnética cardíaca (imagem estática de um cine, eixo curto) confirmando a presença de volumoso pseudoaneurisma ventricular esquerdo (seta a tracejado). VD: ventrículo direito; VE: ventrículo esquerdo.

Mulher, 87 anos, com antecedentes pessoais relevantes de dislipidemia, bócio multinodular e quisto renal direito, recorreu ao Serviço de Urgência (SU) por quadro clínico, com 3 semanas de evolução, caracterizado por cansaço fácil e dispneia para pequenos esforços, pré-cordialgia com irradiação dorsal, tipo moinha, anorexia e náuseas. Encontrava-se hemodinamicamente estável, com fervores bibasais, sem outras alterações assinaláveis ao exame físico. O electrocardiograma mostrava supra-desnivelamento do segmento ST nas derivações DII, DIII e aVF. Laboratorialmente com elevação do valor da troponina I (551,1 ng/L) e NT-proBNP (12.568 pg/mL). A doente foi internada com o diagnóstico de IAM com elevação do segmento ST (IAMcST) inferior. Tendo em conta o tempo de evolução, considerou-se não ter indicação para fibrinólise. O ETT mostrou disfunção biventricular (fração de ejeção do VE de 40% por método Simpson Biplano), acinésia médio-basal póstero-lateral e inferior com formação aneurismática ( Figura 1 ), insuficiência mitral moderada e hipertensão arterial pulmonar moderada. Realizou teste de isquemia (cintilografia de perfusão do miocárdio) sem evidência de isquemia, documentando defeito fixo na parede inferior, não sendo candidata a coronariografia. A doente teve alta com estabilidade clínica e medicada com dupla antiagregação plaquetar, estatina e beta-bloqueante (baixa dose). Passados dois dias regressou ao SU com clínica sugestiva de insuficiência cardíaca. A doente estava taquicárdica, polipneica, com necessidade de aporte suplementar de oxigênio. Radiologicamente visualizava-se derrame pleural bilateral. Eletrocardiograficamente sem alterações dinâmicas. Repetiu-se o ETT observando-se derrame pericárdico moderado, sem sinais de compromisso hemodinâmico, e aumento das dimensões do aneurisma, colocando-se a possibilidade de tratar-se de um PA ( Figura 2 ). Fez RMC em outra instituição ( Figuras 3 e 4 ) que confirmou tratar-se de PA da parede inferior do ventrículo com 7x5,4 cm, de colo largo (3,5 cm), com trombo parietal. O caso foi discutido com a equipe de cirurgia cardiotorácica, que tendo em conta a idade avançada, estado de fragilidade e irreversibilidade do quadro clínico, considerou que a doente apresentava elevada morbi-mortalidade intra e peri-operatória, não beneficiando de tratamento cirúrgico. A doente evoluiu em choque cardiogénico, vindo a falecer após quatro dias de internamento.

**Figura 4:**
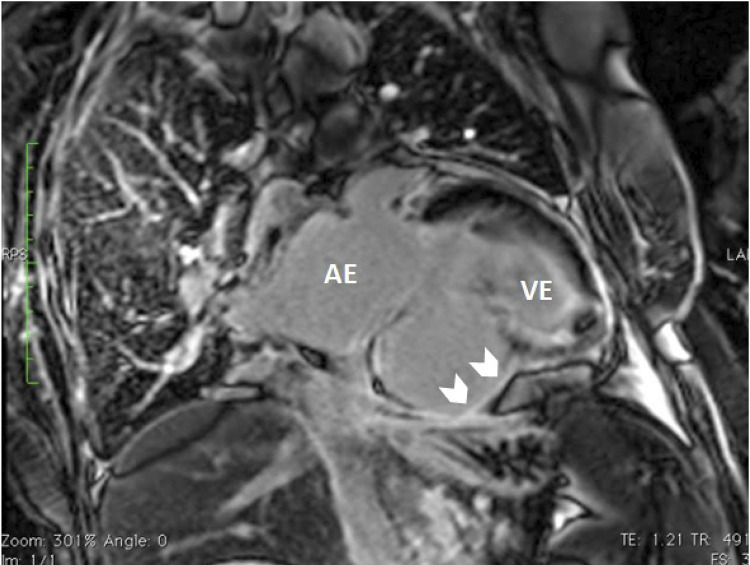
Ressonância magnética cardíaca, após injecção de gadolíneo, observando-se a presença de realce tardio sobre os folhetos pericárdicos (pontas de seta), apoiando o diagnóstico de pseudoaneurisma. AE: aurícula esquerda; VE: ventrículo esquerdo.
