# Supplementary material for: Infarto do Miocárdio Inferior Evoluído com Pseudoaneurisma do Ventrículo Esquerdo: Um Dilema Diagnóstico
Source: Arq Bras Cardiol. 2020 May 12;114(4):730–1. [Article in Portuguese] doi: 10.36660/abc.20200029 (PMC9744350; doi:10.36660/abc.20200029)
Supplement: Translation

# Translation: Evolved Inferior Wall Myocardial Infarction with Left Ventricular Pseudoaneurysm: A Diagnostic Dilemma

**Authors:** Sónia Gomes Coelho, Clara F. Jorge, Pedro B. Carlos, Anne Delgado, Leopoldina Vicente

**Affiliations:** 1 Portugal Centro Hospitalar Cova da Beira EPE - Department of Internal Medicine, Covilhã - Portugal; 2 Portugal Centro Hospitalar Cova da Beira EPE - Department of Cardiology, Covilhã - Portugal

**Keywords:** Myocardial Infarction/complications, Pseudoaneurysm, Heart Rupture, Echocardiography/methods, Magnetic Resonance Spectroscopy/methods

## Introduction

The left ventricular (LV) pseudoaneurysm (PA) is a rare mechanical complication of acute myocardial infarction (AMI).^1^ It results from myocardial rupture, in which the hemorrhagic process is contained by the adherent pericardium. It occurs most commonly in the inferior and posterior ventricular wall, since the rupture of the anterior ventricular wall usually leads to cardiac tamponade and immediate death, while the inferior-posterior face of the heart rests on the diaphragm, facilitating the ventricular cavity containment by the pericardium.^1 - 3^ Imaging methods are crucial to establish the diagnosis. Transthoracic (TTE) and transesophageal echocardiography (TEE) allow the definitive diagnosis in 26% and 75% of cases, respectively.^1 , 2^ Cardiac magnetic resonance (CMR) imaging is useful in the differential diagnosis of LV PA and aneurysm, with a reported sensitivity of 100%.^2^ The presence of late pericardial enhancement in the CMR is highly suggestive of LV PA, which may represent the effect of the passage of blood into the pericardial space at the time of myocardial rupture, with subsequent pericardial inflammation and fibrosis.^1 , 2 , 4^

## Case Report

An 87-year-old female patient, with a relevant personal history of dyslipidemia, multinodular goiter and right renal cyst, came to the Emergency Department (ED) due to clinical symptoms, with 3 weeks of evolution, characterized by frequent tiredness and dyspnea at small efforts, mild and persistent precordial pain with dorsal irradiation, anorexia and nausea. She was hemodynamically stable, had bilateral rales, and no other significant alterations on physical examination. The electrocardiogram showed ST-segment elevation at the DII, DIII and aVF leads. Laboratory tests showed increased troponin I (551.1 ng/L) and NT-proBNP (12,568 pg/mL) levels. The patient was admitted with the diagnosis of AMI with lower ST-segment elevation (STEMI). Taking into account the time of evolution, the case was considered as having no indication for fibrinolysis. The TTE showed biventricular dysfunction (LV ejection fraction of 40% by the Simpson Biplane method), posterolateral and lower mid-basal akinesia with aneurysmal formation ( Figure 1 ), moderate mitral regurgitation and moderate pulmonary arterial hypertension. She was submitted to an ischemia test (myocardial perfusion scintigraphy) with no evidence of ischemia, but a fixed defect was documented in the lower wall, thus not being a candidate for coronary angiography. The patient was discharged under clinical stability and treated with dual antiplatelet therapy, statin and beta-blocker (low dose). Two days later, she returned to the ED with clinical signs suggestive of heart failure. The patient had tachycardia, polypneia, and required supplemental oxygen therapy. Radiologically, bilateral pleural effusion was visualized. The ECG showed no dynamic alterations. The TTE was repeated, showing moderate pericardial effusion, with no signs of hemodynamic compromise, and an increase in the aneurysm size, raising the possibility of its being a PA ( Figure 2 ). She underwent CMR in another institution ( Figures 3 and 4 ), which confirmed that it was 7x5.4 cm lower ventricular wall PA, with a wide neck (3.5 cm), and a parietal thrombus. The case was discussed with the Cardiothoracic Surgery team, which, taking into account the patient’s advanced age, state of fragility and clinical picture irreversibility, considered that the patient had high intra- and perioperative morbidity and mortality, and thus would not benefit from surgical treatment. The patient developed cardiogenic shock and died after four days of hospitalization.

**Figure 1:** Transthoracic echocardiography (apical 2-chamber view) showing aneurysmal formation (arrow).

**Figure 2:** Transthoracic echocardiography (short axis view) suggestive of a pseudoaneurysm of the lower left ventricular wall (asterisk).

**Figure 3:** Cardiac magnetic resonance imaging (static cine image, short axis) confirming the presence of a large left ventricular pseudoaneurysm (dashed arrow). RV, right ventricle; LV, left ventricle.

**Figure 4:** Cardiac magnetic resonance imaging, after gadolinium injection, showing the presence of late enhancement over the pericardial leaflets (arrowheads), supporting the diagnosis of pseudoaneurysm. LA, left atrium; LV, left ventricle.
